# Macrophages in Noise-Exposed Cochlea: Changes, Regulation and the Potential Role

**DOI:** 10.14336/AD.2019.0723

**Published:** 2020-02-01

**Authors:** Weiwei He, Jintao Yu, Yu Sun, Weijia Kong

**Affiliations:** Department of Otorhinolaryngology, Union Hospital, Tongji Medical College, Huazhong University of Science and Technology, Wuhan, 430022, China.

**Keywords:** macrophage, noise induced hearing loss, inflammatory molecule, immune response

## Abstract

Acoustic trauma is an important physical factor leading to cochlear damage and hearing impairments. Inflammation responds to this kind of cochlear damage stress. Macrophages, the major innate immune cells in the cochlea, are important drivers of inflammatory and tissue repair responses after cochlear injury. Recently, studies have shown that after noise exposure, the distribution, phenotype, and the number of cochlear macrophages have significantly changed, and the local environmental factors that shape macrophage differentiation and behavior are also drastically altered. However, the exact role of these immune cells in the cochlea after acoustic injury remains unknown. Here we review the properties of cochlear macrophages both under steady-state conditions and non-homeostatic conditions after cochlear acoustic injury and discuss their potential role in noise-exposed cochlea.

## 1.Introduction

The cochleae, as a connection of the middle ear and central nervous system (CNS), converts mechanical stimuli into nerve impulse in the auditory system. Cochlear hair cells (HCs) with surrounding supporting cells, reside on the top of the basilar membrane. HCs come in two types, the outer hair cells (OHCs) and the inner hair cells (IHCs). OHCs amplify input sound energy, and IHCs transmit sound stimuli to the CNS though spiral ganglion neurons (SGNs). Noise-induced hearing loss (NIHL) was mainly attributed to loss of cochlear hair cells and spiral ganglion neurons caused by excessive noise exposure. However, the cellular and molecular mechanisms underlying NIHL have not been fully clarified, therefore, clinically NIHL lacks effective therapeutic methods. Recently, inflammation and immune response have been increasingly recognized as important pathophysiological mechanisms underlying irreparable or reparable damage to hair cells and neurons after acoustic injury[1-3]. As the major professional immune cells in the cochlea, macrophages contribute to the inflammation, repair, and homeostasis, playing a critical role in local cochlear immunity[4]. Macrophages fulfill their immunological functions in direct and indirect ways, by engulfing foreign pathogens, dead cells and cellular debris, producing proinflammatory cytokines and chemokines, and participating in immunoregulation. Their properties and functions are mainly determined by local environmental factors, which allows them to adapt highly to the needs of their anatomical niche. Studies have shown that after noise exposure, the distribution, phenotype, and the number of cochlear macrophages have significantly changed, and the local environmental factors that shape macrophage differentiation and behavior are also drastically altered, which supports the concept that macrophages play a vital role in cochlear response to acoustic injury[3, 5]. However, the exact role of these immune cells in the cochlea after noise exposure remains unknown. To understand the functions of cochlear macrophages after acoustic trauma, in this review, we review the properties of cochlear macrophages both under steady-state conditions and inflammatory conditions after cochlear acoustic injury, and try to figure out what happened to these cells and discuss their potential role in noise-exposed cochlea.

## 2. Macrophages in the steady-state cochlea

### 2.1 Distribution of cochlear resting macrophages

Under steady-state conditions, cochlear macrophages are found in multiple cochlear regions, including the spiral ligament, spiral limbus, spiral ganglion region, osseous spiral lamina, basilar membrane, and stria vascularis [1, 6-9]. A large population of macrophages reside in the cochlear lateral wall and the spiral ganglion area. But in the spaces of the scala media and organ of Corti macrophages are notably absent [1].

In the basilar membrane, under steady-state conditions, macrophages are occasionally identified on the scala tympani side from the apical to the basal portion. Morphology of macrophages varies in different regions of the basilar membrane. Apical sections present with ramified, dendritic macrophages. In the middle region, macrophages display dendritic-to-amoeboid transitionary phenotype. Basal portions are dominated by macrophages with amoeboid morphology [2, 10]. In addition to morphological characteristics, apical and basal macrophages also have different expression patterns for immune proteins [2].

In the cochlear lateral wall, macrophages are distributed both in the spiral ligament and stria vascularis, with different phenotypes and functions. In the spiral ligament, macrophages are observed to be more abundant in the lower portion, corresponding to the type II and IV ﬁbrocyte regions, and these cells show irregular shapes with branches and processes and share the same characteristics with general macrophages [11]. In the stria vascularis, macrophages are dendrite-shaped with ramified processes distributed in the surroundings of stria capillaries, and display characteristics of both melanocytes and macrophages, which have been shown to be responsible for the integrity of the cochlear intrastrial fluid-blood barrier [9, 12].

In the organ of Corti, it has been observed that there are no macrophage under steady-state conditions in the mature organ of Corti [1, 2, 7, 13]. However, recent evidence showed that the postnatal organ of Corti contains macrophages. Dong et al detected irregular-shape macrophages on the organ of Corti side of the basilar membrane at postnatal day 1 (P1) in mice. But macrophages gradually display shrunken cell bodies and fragmented or shrunken nuclei beginning from the basal region and progressing to the apical region starting from P7. Finally, all macrophages disappeared, leaving anucleated residual bodies with the maturation of the sensory epithelium observed at P17-21[14].

In the cochlear nervous system, macrophages are close to nerve fibers in the spiral lamina and in the modiolus under steady-state conditions. And macrophages in these areas may participate in maturation of the auditory nerve and hearing onset. For example, cochlear macrophages eliminate supernumerary glial cells during auditory nerve development, and depletion of macrophages results in elevated glial cell numbers, abnormal myelin sheath formation, and diminishing hearing functions [15].

### 2.2 Origin of cochlear macrophage under steady-state conditions

In peripheral tissues, the tissue-resident macrophages are maintained by different precursor populations that can be recruited from embryonic haematopoietic precursors, bone marrow-derived myeloid precursors and self-renewal. In the inner ear, during the development, embryonic macrophages have been observed associated with the otic vesicle at embryonic day 10, indicating the presence of embryonic origin of cochlear macrophages [16]. And after birth, during the postnatal development, immune cells with macrophage precursor phenotypes were also detected in the cochlear basilar membrane and these cells can locally differentiate into mature macrophages[14]. In adult animals, tissue-resident macrophages are fully differentiated cells that have lost proliferative potential and are constantly repopulated by circulating monocytes produced by bone marrow-derived myeloid progenitors. Using transgenic homozygous mice for green fluorescent protein (GFP) as donor, Tan et al transplanted bone marrow of donor to wild-type recipient mice. Thus, after bone marrow transplantation (BMT), bone marrow-derived cells in recipient mice display GFP-positive. Three months after BMT, GFP-positive cells were observed in the cochlea and most of them were CD45 and CD68 positive, which demonstrated that bone marrow-derived macrophages are at least one way of the origin of cochlear macrophages in adult mice[17] . These results indicate that cochlear macrophages with different ontological origins coexist in the inner ear.

In peripheral tissue, resident macrophages play a role as supervisors and contribute to inflammation in the early stage of tissue damage. In some tissues resident macrophages also have tissue-specific functions and different activation conditions to diverse stress. In the cochlea, the distribution of macrophages under steady-state conditions is unambiguous, but the characterization of these macrophages requires additional exploration. Whether these cochlear resting macrophages have specific functions that are divergent from circulating monocytes, or whether there are significant differences between cochlear macrophages under steady conditions and infiltrated macrophages after damage is unknown.

## 3. Macrophages in the noise exposed cochlea

After acoustic injury, loss of cochlear hair cells by physical and mechanical forces is regarded as the initial damage. And the secondary damage develops hours and days after noise exposure, including excitotoxicity, oxidative stress, and widespread inflammation. In cochlear inflammation, the death of hair cells and production of molecules by resident cells help the activation and recruitment of macrophages. Then activated resident macrophages and monocyte-derived macrophages induce and magnify the inflammatory responses by secretion of molecules. On the one hand, molecular responses and phagocytosis of macrophages may detrimental to cochlear tissues, causing expanding lesions and further dysfunctions. On the other hand, these cells also work as scavengers and aid in healing. Here, we focus on macrophages responses after acoustic trauma.

### 3.1 Distribution changes of cochlear macrophages after noise exposure

**Figure 1.**
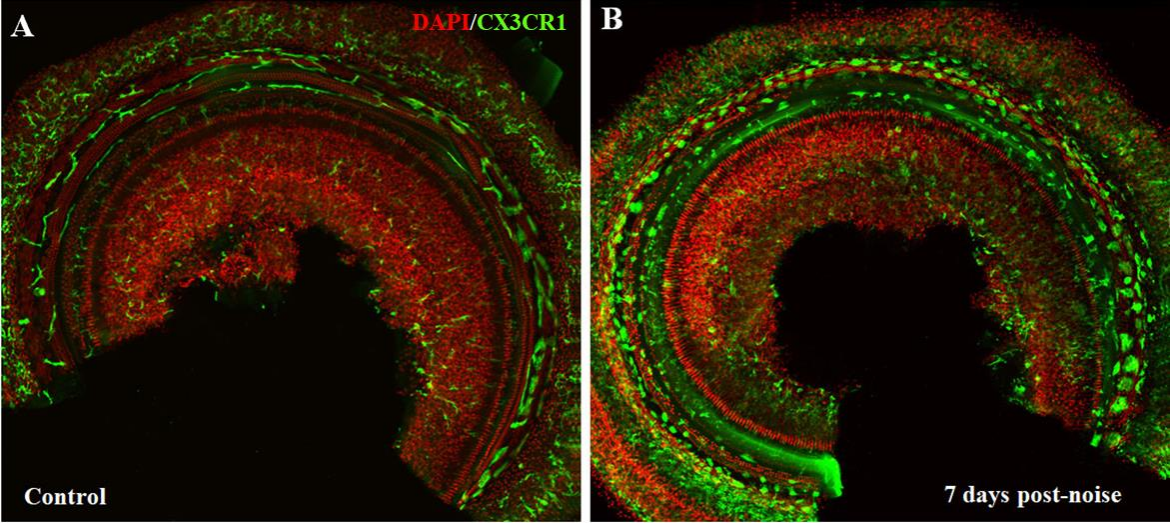
**Macrophage changes after noise exposure in mouse cochlea (CX3CR1^+/GFP^ transgenic mice generated on a CBA background)**. **(A)** In control cochlea, macrophages were relatively rare with ramified, dendritic phenotypes. **(B)** Seven days after noise exposure (110dB, white noise for 2 hours), cochlear macrophages were significantly increased with amoeboid morphology.

Intense acoustic overstimulation leads to a significant increase of leukocytes in the cochlea. These leukocytes, particularly circulating monocytes enter cochlea, differentiate into mature macrophages and respond to tissue damage (Fig. 1). Several groups have reported the peak level of macrophages appears to occur between 3 and 7 days after acoustic trauma [1, 2, 18-20].

The increased macrophages have been observed in various sites of the cochlea, especially in the spiral ligament and the spiral limbus [1, 19, 21-23], where fibrocyte loss has been demonstrated after noise damage. The increase is also found in the basilar membrane, spiral ganglion area and fluid-filled spaces of the scala tympani and scala vestibuli [1, 19-21, 23]. In the modiolus and the spiral ganglion area, the number of macrophages is significantly decreased at 24 h after noise exposure, then appear to increase [24]. The temporary decrease may result from migration of resident macrophages from spiral ganglion toward the spiral lamina and epithelium. Infiltration and accumulation of bone marrow-derived cells is significant following acoustic trauma [17]. These bone marrow-derived cells, mostly identified as macrophages, peak at 3 days post noise exposure and are most prominent in the spiral ligament and spiral limbus, consistent with the distribution of increased macrophages [17]. Hirose et al provided evidence that there is little proliferation of cochlear macrophages after noise [1]. These data support that circulating monocytes and macrophages migrate into the cochlea through the vasculature of the lateral wall, modiolus and ganglion after noise exposure.

### 3.2 Molecular regulations of cochlear macrophages after acoustic trauma

Determining the interaction of molecules and macrophages in the cochlea after noise injury is a vital step to understand the extracellular regulation of macrophages. In peripheral tissues, molecules and macrophages participate in the mobilisation and amplification of inflammatory responses. Initially, stress stimulates endothelial cells to present cellular adhesion molecules that recruit macrophages to the damaged tissue. Adhesion molecules make macrophages slow down and adhere to endothelium, then change phenotype and permeability of endothelium to allow macrophages to move toward where resident cells secrete cytokines. Within the tissue, monocytes differentiate into macrophages capable of phagocytosis and secretion of further molecules, mobilising further immune responses.

Toll-like receptor 4(Tlr4), a membrane receptor, is a member of the pattern recognition receptor (PRR) family. Tlr4 recognizes pathogen- or damage- associated molecular patterns (PAMPs or DAMPs) that are expressed on infectious agents or damaged tissues and contribute to the initiation of immune responses. In the cochlea, Tlr4 is constitutively expressed in the sensory epithelium and acoustic overstimulation causes Tlr4 to be upregulated, but Tlr4 knockout does not affect the hearing functions under normal conditions [13]. After acoustic injury, Tlr4 knockout mice are relatively noise-resistant compared to control mice. Absence of Tlr4 inhibits macrophages to express major histocompatibility complex class II (MHC- II), reducing antigen-presenting activity of macrophages. Dysfunction of Tlr4 suppresses the production of IL-6 in the organ of Corti, but not in the lateral wall and basilar membrane [25].

ICAM-1 is an adhesion molecule and mediates adhesion of leukocytes to vascular endothelial cells in inflammatory responses. In normal cochlea, ICAM-1 is present on the vascular endothelial cells [26] and upregulates after noise exposure. After acoustic trauma, the amount of ICAM-1 expression is slightly increased in the spiral ligament, with protein expression beginning at 24 h post noise, reaching a maximum at 2 and 4 days and returning to basal levels by 14 days [19]. Additionally, blocking of ICAM can protect against noise-induced hearing loss [27]. However, whether this protective effect on noise-induced hearing loss is associated with the inhibition of macrophage recruitment remains unknown.

In the regulation of cochlear responses to acoustic stimulation, the proinflammatory cytokines, including tumor necrosis factor alpha (TNF-α), interleukin-1beta (IL-1β), and interleukin-6 (IL-6), are detected in the early phase of noise stimulated cochlea in previous studies. These cytokines are produced by activated macrophages, are important mediators of the acute phase of inflammatory responses and are involved in a variety of cellular activities. IL-1β and IL-6 are significantly increased in the early stage after noise exposure, but quickly downregulate to the baseline [20, 28]. IL-6 expression is observed in the spiral ligament, stria vascularis, and spiral ganglion neurons [28]. Wakabayashi et al found that in noise-exposed mice, ABR thresholds of low frequencies was improved by blocking the IL-6 signaling [20]. Also, inhibiting the IL-6 signaling suppresses the infiltration of cochlear macrophages in the spiral ganglion, but not in the lateral walls. TNF-α induces the recruitment of inflammatory cells into the cochlea [29]. But the IL-1β-/- mice do not exhibit significant reduced threshold shifts at any examined frequency, compared to wild-type mice [30]. Inhibition of IL-1β does not reverse noise-induced cochlear damage, and there is no proof that IL-1β can influence macrophages entering the cochlea.

Chemokines are the largest family of cytokines, and this family can be divided into four subclasses: C, CC, CXC, and CX3C. The CC and CXC chemokines are the major subgroups, and the C and CX3C chemokines are much smaller with only a few members. The CC chemokines are primarily chemotactic for monocytes/ macrophages [31], and the CXC chemokines are potent inducers of neutrophil activation and migration [32]. Here we discuss chemokine CCL2, fractalkine (CX3CL1) and their receptors in cochlea. Following acoustic trauma, increase of CCL12 and CCL2 occur very early as determined by gene array analysis and RT-PCR [19]. The ability of CCL2 is to activate and attract monocyte lineage cells including macrophages, monocytes, and microglia in system immune responses. Mice deficient in CCL2 are unable to effectively recruit monocytes [33], and in the CNS, targeted CCL2 overexpression of transgenic mice results in the accumulation of macrophages [34]. However, in the cochlea, Sautter et al observed that monocyte migration was unchanged, despite the absence of CCL2 or CCR2, and the CCR2, independent of CCL2, plays a protective role in the cochlea after noise[23]. Fractalkine, also known as CX3CL1, the only member of the CX3C chemokine family, is expressed at high levels by neurons and collaborates with its receptor to mediate attraction of immune cells. The fractalkine receptor (CX3CR1) is expressed on monocytes, tissue macrophages, natural killer (NK) cells, activated T cells, and microglia. In the cochlea, there was no significant difference in hearing thresholds, accumulation of macrophages, and tissue injuries after noise exposure between absence and presence of CX3CR1 in mice [8]. Lack of CX3CR1 has no effect on noise-induced recruitment of macrophages, but results in increased loss of SGNs and enhances expression of the inflammatory cytokine IL-1β [24]. In the model of selective hair cell lesion, disruption of fractalkine signaling reduces macrophage recruitment into spiral ganglion and diminishes survival of SGNs [35]. For a group of atypical chemokine receptor, Duffy antigen receptor for chemokines (DARC) is essential for chemokine-regulated inflammatory cells trafficking. Darc-KO mice exhibited improved hearing recovery after intense noise exposure when compared to control mice[36], but the potential relationship between DARC and cochlear macrophages after noise require additional studies.

Taken together, these results indicate that many molecules influence the cochlear functions by increasing or decreasing their expression. But there is no study demonstrating that any molecule signaling significantly regulate the recruitment of macrophages alone. The infiltration of monocytes is a response of “teamwork” of various factors. The peak level of the inflammatory cytokines occurs in early stage after acoustic injury, compared with the level of macrophage, which achieve a peak post 3-7 days. This discrepancy suggests that the inflammatory mediators produced in early stage is rarely came from infiltrated macrophages. Instead, cochlear fibrocytes and tissue macrophages may contribute to early cochlear immune response by releasing cytokines and chemokines.

## 4. Functions of macrophages in noise-damaged stria vascularis

In the stria vascularis, the macrophage is not only responsible for immune functions, but also has an important role in regulating the integrity and permeability of the blood-labyrinth-barrier [12, 37]. The cochlear blood-labyrinth-barrier (CBLB) separates the stria vascularis from peripheral circulation and maintains inner ear homeostasis, and is constituted by endothelial cells, pericytes, basement membrane, tight and adherens junctions and strial macrophages.

Acoustic trauma leads to a dramatic change in cochlear blood flow and disrupts the CBLB [38, 39]. After sufficient noise exposure, shifting of pericytes, injury of endothelial cells, decrease of junction proteins and activation of strial macrophages all contribute to an increase of vascular permeability [39-41]. Activated strial macrophages become smaller with shorter processes and physically detachment from capillary walls [41], and produce less pigment epithelial-derived factor which is crucial for stabilizing the CBLB, and maintaining normal hearing functions [12, 42].

Strial macrophages are renewed by migration of infiltrated macrophages to the CBLB, noise exposure accelerates the circulating of peripheral macrophages [9, 43]. Using a mouse model with transplantation of bone marrow-derived macrophage, Dai et al showed that bone marrow-derived macrophages migration occurred in the area of injured CBLB after noise treatment, and these infiltrated macrophages were gradually elongated in morphology and distributed parallel to the vessels of the stria vascularis, where they differentiate into mature strial macrophages and aid in cochlear vascular repair [43].

Taken together, distinct from macrophages in other cochlear sites, strial macrophages change the CBLB by not only immune responses but also physical and molecular dysfunctions. Regulation strial macrophages and their functions may be a future target for clinically interventions in the amelioration of noise-induced hearing loss.

## 5. Noise-induced inflammatory responses in the organ of Corti

Generally, in the cochlea, migrating and resident immune cells respond to acoustic stress. However, as we mentioned above, this kind of response only occurs in the non-organ of Corti tissues. The organ of Corti is not mediated by immune cells, but the resident cells in the organ of Corti have immune capacity.

The organ of Corti contains sensory cells and supporting cells, the sensory cells are susceptible to stress, and the supporting cells maintain the functional and structural integrity of the organ of Corti. Several studies have reported that the supporting cells express immune genes[44-46]. Recently, using high-throughput RNA-sequencing and qRT-PCR arrays, Cai et al provided evidence of robust expression of immune and inflammatory genes in the organ of Corti, and that these genes could respond to acoustic stress. In their study, supporting cells are the primary response site and outer hair cells have low immune activity [13]. Supporting cells, such as Deiter cells express Tlr4 and have a phagocytic activity to engulf sensory cell debris; also they express many immune genes after noise exposure [13, 47]. Additionally, Deiter cells maintain the ion composition of the endolymph and the organ of Corti’s lymph by expanding the phalangeal processes to seal defects in the reticular lamina [48].

## 6. Summary

Circulating monocytes enter the cochlea after noise exposure, where they differentiate into mature macrophages and participate in immune responses together with tissue macrophages. During this process, many inflammatory mediators significantly increase early, playing an essential role in hearing loss and infiltration of macrophages. These cytokines and chemokines regulate the recruitment of macrophages, and also are responsible for noise-induced cochlear inflammation. In noise-treated cochleae, the function of macrophages is not clear, and there is limited knowledge regarding how these cells work after entering and activating. Understanding the role of macrophages in acoustic trauma may provide more possibility to figure out the mechanism of noise-induced damage.

Firstly, about the immune functions of peripheral macrophage, Mills and Leyb conclude as “SHIP”: sample, heal, inhibit and present [49]. For sampling, the surface of macrophages processes two basic types of sampling receptor to analyze surroundings: damage- and pathogen-associated molecular patterns (DAMPs and PAMPs). Macrophages, via their “heal” mode, remove debris or dead cells. They can also switch to the “inhibit” mode and produce chemicals to kill enemy pathogens. Macrophages can be divided into M1 or M2 activation by its functions of “inhibit” and “heal”. M1 macrophages produce an aggressive immune response. M2 macrophages have roles in wound healing and regulation of the macrophage response. The fourth function is the ability to “present (antigen)” and initiate adaptive immunity. In further study, polarization and function of cochlear macrophages probably are new feasibilities to explain their role in noise-induced damage.

Secondly, circulating monocytes and tissue macrophages are both mononuclear immune cells, with similar morphology, common origin and overlapping immune functions. Under steady-state conditions, mononuclear cells have common phagocytic abilities across different tissues [50], but tissue-resident macrophages share expression of only a few unique transcripts, with most expressions being specific to the resident tissue [51, 52]. For example, bone osteoclasts, brain microglia, liver Kupffer cells and lung alveolar macrophages display their own specific purpose [53-57]. In the cochlea, Brown et al found that macrophages play a critical role in nerve refinement of SGNs in the developing auditory nerve[15], which is similar with microglia in the central nervous system [54, 58]. In future studies, more specific functions of cochlear macrophages under steady conditions and difference between cochlear resident macrophages and circulating infiltrated macrophages need more exploration. In short, to clarify the origin and functions of cochlear macrophages will may be one of the key steps to reveal new targets for preventative therapies in noise-induced hearing loss.

Moreover, the immune response not only works in acute cochlear damage, as some studies in other tissues have advanced the notion that inflammation as a mechanism of aging and age-related pathogenesis. In the cochlea, aging increases the risk of noise-induced cochlear damage and induces deterioration of macrophages in quantity and quality. In aging mice, cochlear macrophages display enlarged cell bodies and a grainy appearance with irregular-shaped nuclei. Also, the average size of macrophages become significantly smaller [10]. In the aging stria vascularis, some macrophages are flat and amoeboid-shaped, with less physical contact to strial capillaries [59]. Whether macrophages could directly or indirectly influence age-related hearing loss (AHL) or whether there are some interactions between aging and cochlear inflammation may provide an avenue to understand the relationship between acoustic trauma and aging.
